# Genome-wide association studies with prolapsed gland of the third eyelid in dogs

**DOI:** 10.3389/fvets.2024.1520155

**Published:** 2025-01-24

**Authors:** Yu Zeng, Cundong Feng, Zheli Jiang, Weian Du, Shan He, Xingnuan Li, Yi Fan, Xiao Ouyang, Bixin Huang, Yan Su, Siyu Wang, Rongxing Wei, Zonghao Dai, Peng Jin, Jianyun Liu, Qianyong Yang

**Affiliations:** ^1^Jiujiang Key Laboratory of Rare Disease Research, Jiujiang University, Jiujiang, China; ^2^Jiangxi Provincial Key Laboratory of Cell Precision Therapy, School of Basic Medical Sciences, Jiujiang University, Jiujiang, China; ^3^Jiujiang Innovation Center of Biosensor Technology and Application, Jiujiang University, Jiujiang, China; ^4^School of Tropical Agriculture and Forestry (School of Agricultural and Rural Affairs, School of Rural Revitalization), Hainan University, Haikou, China; ^5^School of Stomatology and Medicine, Foshan University, Foshan, China; ^6^Nanchang Police Dog Base of the Ministry of Public Security of China, Nanchang, China

**Keywords:** cherry eye, third eyelid gland, animal model, canine, eye diseases, genetics

## Abstract

Cherry eye, the common name for the prolapse of the third eyelid gland in dogs, is a widespread ophthalmic disease affecting dogs of various breeds. This condition severely affects the quality of life of affected dogs, and its underlying cause remains unresolved. In this study, 170K SNP microarray data were collected from 653 brachycephalic dogs and 788 brachycephalic and mesocephalic dogs. These two datasets were analyzed separately in genome-wide association studies (GWAS) involving 12 dog breeds affected by cherry eye. The GWAS analysis of 653 short-headed dogs revealed that four SNPs in the CFA3:15627075-15983629 bp region exceeded the genome-level significance threshold. Association analysis of this region also indicated that these four SNPs were strongly associated. Gene annotation showed that the region contained genes such as *KIAA0825, FAM172A*, and *NR2F1*, of which *NR2F1* was associated with eye development. The results showed that GWAS analysis performed on 788 short- and medium-headed dogs identified five SNPs in the CFA22:15627075-15983629 bp region that exceeded the genome-level significance threshold, and association analysis was performed in this region, which showed that these five SNPs were strongly associated. In addition, 104 annotated genes were identified in both GWAS. To explore the genes involved in cherry eyes, we performed GO functional enrichment analysis. The genes involved in the high pathway were *DIO3* and *TTC8*. In addition, an in-depth analysis revealed 33 genes associated with eye development and diseases. Our study provides new perspectives for further understanding cherry eye in dogs.

## 1 Introduction

**Figure 1:**
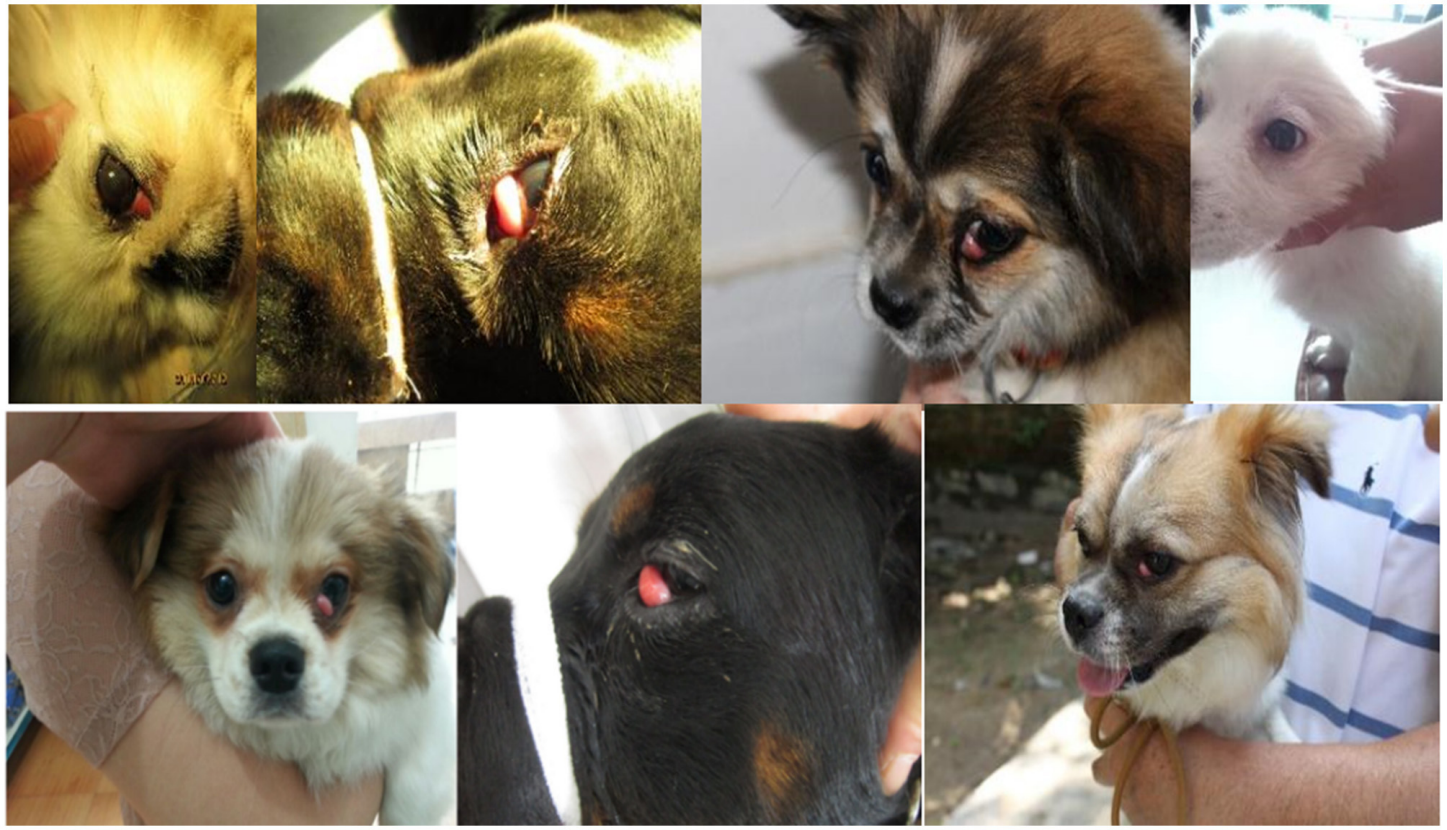
Dogs third eyelid hyperplasia case.

Prolapse of the third eyelid gland in dogs, characterized by a protuberance of the gland at the free edge of the papillae, results in hypertrophy, hyperplasia, or adenoma, forming a prominent red fleshy mass, often referred to as the cherry eye (Figure 1) (1). The cherry eye may be large, covering a large portion of the cornea, or it may be small and appear only periodically (2). Dogs have three eyelids, two of which are easily visible, and an additional eyelid, the third eyelid, which is usually hidden below the inner corner of the eye (2, 3). The third meibomian gland is located at the base of the T-shaped hyaline cartilage in the roughly triangular conjunctival fold at the corner of the eye and it produces 30–60% of tears (4, 5).

The cause of cherry eyes is currently unknown and is hypothesized to be the laxity of the connective tissue that anchors the third eyelid gland, usually on a genetic basis (6–8). Some researchers have suggested that gland enlargement (lymphoid hyperplasia) is caused by antigenic stimulation due to the exposure of puppies to environmental allergens (5, 7, 9). It can be caused by dysplasia or congenital defects in all current breeds but is more common in brachycephalic dogs, such as American and English Cocker Spaniels, Boston Terriers, Peking Pugs, Beagles, and Bulldogs (8). This is mainly because the facial and orbital appearances of brachycephalic dogs are characterized by a rounded cranial shape and flat orbits, which potentially lead to physiological protrusion of the eyeballs, failing to adequately cover the eyeballs, provide lubrication, and make the cornea less sensitive, leading to a variety of ocular disorders (10). Studies have shown that males have a higher incidence than females and the age of onset is between 3 and 12 months (6, 8, 11). Research has indicated that this disease is most common in dogs that usually eat meat, chicken liver, ham sausage, etc., and are well nourished, which may be due to glandular secretions and sticky glandular excretory ducts caused by poor excretion during the development of complete obstruction (12). Symptomatically, the prolapsed gland grows from small to large slowly in 3–5 days, quickly in 1–2 days, and does not continue to develop after swelling to its maximum size. In most dogs, ocular inflammation is not obvious, and in a few cases, it is mainly caused by ocular discomfort, scratching, or friction from an object. Tumors and inflammation can be ruled out from the limited enlargement of the prolapsed gland and the inflammatory manifestations in the affected eye; however, obstruction of the glandular excretory ducts can explain the above symptoms. The degree of obstruction in the glandular excretory ducts determines the disease course's progression rate, and the gland's histological structure determines the limit to its enlargement (6, 11, 12). Pathophysiologically, the glands are enlarged (6), the follicles are dilated (11), the epithelium of the follicles is detached (11, 12), and the nictitating gland dry (13, 14).

This disease is most common in monocular prolapses, a small number of both eyes prolapse at the same time; there are also eye prolapses after the cure of the prolapse of the other eye, followed by prolapse (11, 13). Dogs with third eyelid glandular hyperplasia are initially enlarged due to infection, and gradually, a red fleshy lump appears at the inner corner of the dog's lower eyelid, which increases in size in a short period (13). Sick dogs have conjunctival redness, tearing, and obvious signs of ocular inflammation and discomfort, as well as a marked increase in ocular discharge. Simultaneously, some sick dogs often scratch their eyes with their front paws; therefore, there is a possibility of mechanical damage to the cornea or aggravation of ocular inflammation, which seriously affects the health and lifespan of domestic dogs. Third eyelid gland prolapse is common in domestic dogs and seriously affects the quality of life of the affected dogs (14).

Cherry eye is usually not painful and does not affect a dog's vision; however, if left untreated, it can cause irritation and inflammation (9). Total glandular excision is commonly used to remove the third meibomian gland or to reposition the gland in domestic dogs affected by cherry eye disease (15). Studies have shown that after the removal of the desmoid gland, there is a decrease in tear production, leading to dry keratoconjunctivitis (KCS) (16). KCS is a chronic inflammatory disease that may result from ductal obstruction and is more likely to occur in females compared to males, mainly due to more intense lymphocytic infiltration of the third oculocutaneous gland, with less secretion (3, 17). KCS recurrence is usually associated with repositioning surgery (6, 18). The current popular treatment is the modified Morgan Pocket technique, which is simpler and less prone to recurrence and complications than total adenomectomy (19, 20).

Although more attention has been paid to the treatment of such diseases in domestic dogs, the genetic background of third eyelid gland prolapse in domestic dogs remains of interest. In this study, we performed a genome-wide association studies (GWAS) to understand the genetic mechanism underlying third eyelid gland prolapse in domestic dogs with ophthalmoplegia.

## 2 Materials and methods

### 2.1 Data

The 12 dogs of different breeds suffering from third eyelid prolapse in the experimental animals came from the Police Dog Hospital at the Nanchang Police Dog Base of the Ministry of Public Security, and these diseased dogs were brought to the hospital by nearby pet breeders, the dog suffering from prolapse of the third eyelid is shown in Figure 1. The breeds of these domestic dogs were Pekingese, Rottweiler, and Butterfly, totaling 12. In this study, we also collected 170 K SNP microarray data from 653 dogs with brachycephalic characteristics (Table 1) and 788 brachycephalic and mesocephalic special dogs (Table 2), such as Newfoundlands, which were previously published as 170K high-density SNP microarray data (21).

**Table 1:** Data of 653 brachycephalic dogs used in this study.

**Breed**	**Origin**	**FamID**	**NO**.	**Data**
Pug	China	CN_BG	18	Article
Tibetan Mastiff	China	CN_TM	22	Article
Lion Dog	China	CN_Shi	13	Article
Chow Chow	China	CN_CC	6	Article
Shih Tzu	China	CN_XiS	27	Article
Lhasa Apso	Xizang, China	CN_LhA	15	Article
Tibetan Terrier	Xizang, China	CN_Tt	7	Article
Chinese Chongqing Dog	Sichuan, China	CN_CDh	12	Self Test
Rottweiler	Germany	LWNGer	7	Self test
Rottweiler	Germany	G_RW	96	Article
Miniature Schnauzer	Germany	G_MiSNR	60	Article
Yorkshire Terrier	Britain	E_YKt	211	Article
Teddy	France	F_TPD	16	Article
Maltese	Republic of Malta	MS_MAL	85	Article
Chihuahua	Mexico	M_CHH	14	Article
Havanese	Cuba	Cu_HaN	44	Article
Total	-	-	653	-

**Table 2:** Data of 788 brachycephalic and mesocephalic dogs used in this study.

**Breed**	**Breed origin**	**FamID**	**NO**.	**Data origin**
Pug	China	CN_BG	18	Article
Tibetan Mastiff	China	CN_TM	22	Article
Shar Pei	China	CN_GDSP	29	Article
Lion Dog	China	CN_Shi	13	Article
Chow Chow	China	CN_CC	6	Article
Shih Tzu	China	CN_XiS	27	Article
Lhasa Apso	Xizang, China	CN_LhA	15	Article
Tibetan Terrier	Xizang, China	CN_Tt	7	Article
Chinese Chongqing Dog	Sichuan, China	CN_CDh	12	Self test
Rottweiler	Germany	LWNGer	7	Self test
Rottweiler	Germany	G_RW	96	Article
Miniature Schnauzer	Germany	G_MiSNR	60	Article
Yorkshire Terrier	Britain	E_YKt	211	Article
Teddy	France	F_TPD	16	Article
Maltese	Republic of Malta	MS_MAL	85	Article
Chihuahua	Mexico	M_CHH	14	Article
Havanese	Cuba	Cu_HaN	44	Article
Newfoundland	Canada	Ca_NFL	106	Article
Total	-	-	788	-

### 2.2 Genomic DNA sample preparation

Blood samples were collected from the place of origin of each local dog, using EDTA-K2 anticoagulation negative pressure tubes, and peripheral venous anticoagulated blood was collected by professional veterinary staff, and couriered to the Nanchang Police Dog Base of the Ministry of Public Security in low-temperature refrigeration using a foam insulated box and preserved at −80°C. All samples were collected following the regulations of the Ministry of Agriculture on the protection of test animals and guidelines for their use.

Genomic DNA was extracted using the SE Blood DNA Kit (OMEGA, USA) kit from OMEGA concerning the instruction manual, and the DNA was dissolved into TE buffer, and the concentration of DNA was determined and quality checked using the Nanodrop-1000 (Thermo Scientific, USA) Nucleic Acid Protein Analyzer, and the qualified The DNA samples were required to have A260/230 ratio of 1.7~1.9 and A260/A280 ratio of 1.8–2.0. After uniformly diluted to 100 ng/μL, agarose gel electrophoresis was performed, which required bright bands and no protein and RNA contamination or DNA degradation and was placed in a −20°C refrigerator for backup for a short period.

### 2.3 Genome-wide association studies

These two datasets were used to perform a GWAS based on a case-control study with 12 cherry-eyed dogs. We set the phenotype of dogs with cherry-eyed dogs with phenotype set to 1 and other dogs to 0. The genomic significance level was set to -log(1/Nsite), where Nsite denotes the number of SNPs, and the highly significant level was -log(1/Nsite)+2. Visualization was performed using R language, and LD block analysis was performed using LDBlockMovie software (21).

## 3 Results and analysis

**Figure 2:**
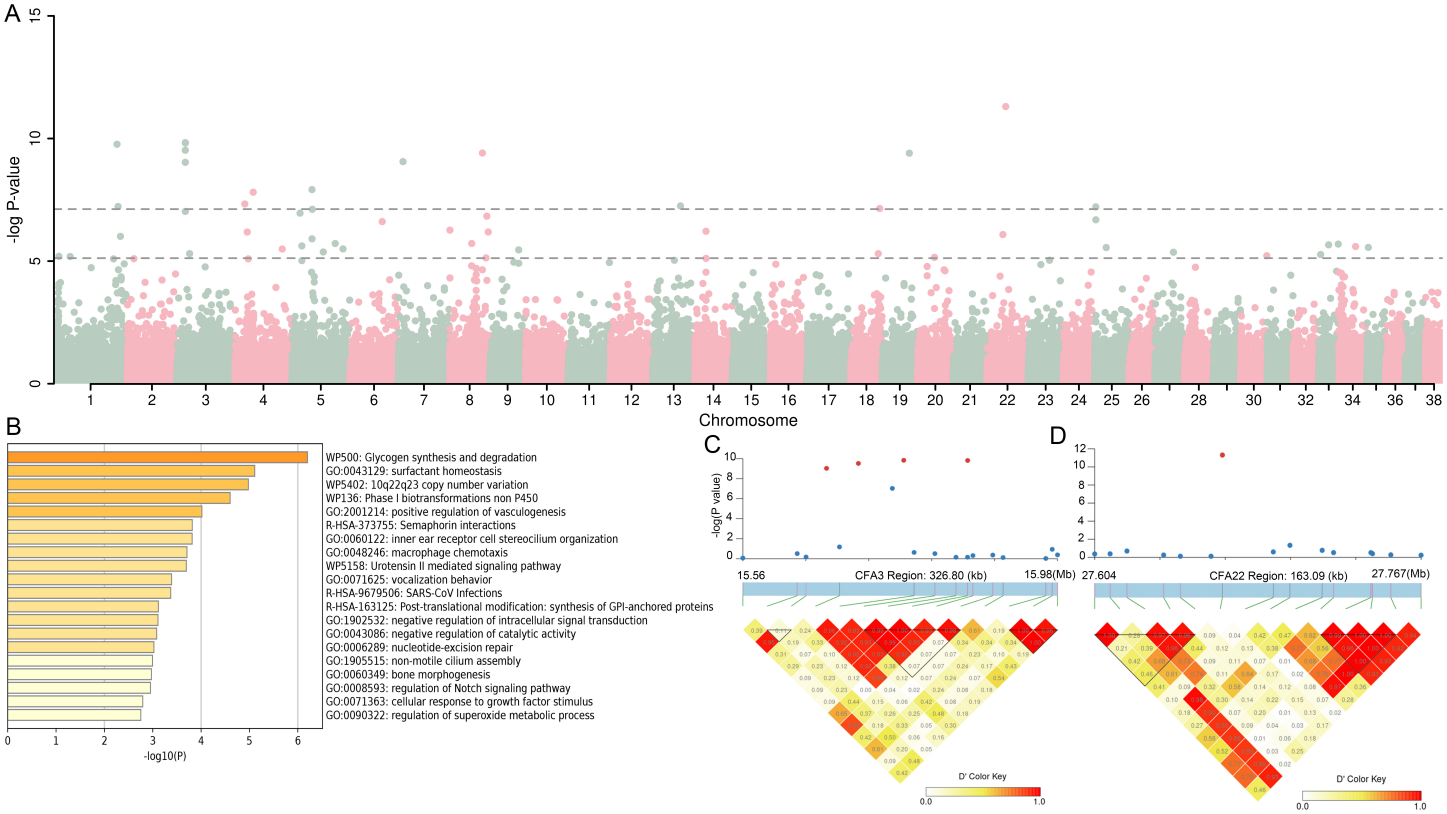
Genomic association analysis (GWAS) between 12 infected dogs and 653 dogs with brachycephalic characteristics. **(A)** Manhattan map of GWAS. **(B)** The function of the 240 genes enrichment analysis. **(C, D)** LD block analysis of the two regions on CFA3 and CFA22.

In this study, GWAS results from 12 dogs with cherry eye compared to 653 brachycephalic dogs showed a total of 52 SNPs exceeding the threshold of significance at the chromosomal level, with 16 SNPs exceeding the threshold of significance at the genomic level (Figure 2A). We genetically annotated SNPs and their upstream and downstream 5Kb regions that exceeded the significance threshold at the chromosome level, and identified a total of 240 genes (Supplementary Table S1), which were analyzed for functional enrichment, and the results showed that these genes were involved in multiple biological functions (Figure 2B). Notably, we identified four SNPs in the CFA3:15627075-15983629 bp region that exceeded the genome-level significance threshold. Chaining analysis of this region revealed that these four SNPs were strongly interlinked (Figure 2C), and gene annotation revealed that this region contained three genes, including *KIAA0825, FAM172A*, and *NR2F1*, of which *NR2F1* is associated with eye development (22). We found an SNP on CFA22 well above the significance threshold, and chaining analysis of this SNP and its upstream and downstream regions showed that this SNP did not appear to be chained to nearby regions (Figure 2D).

**Figure 3:**
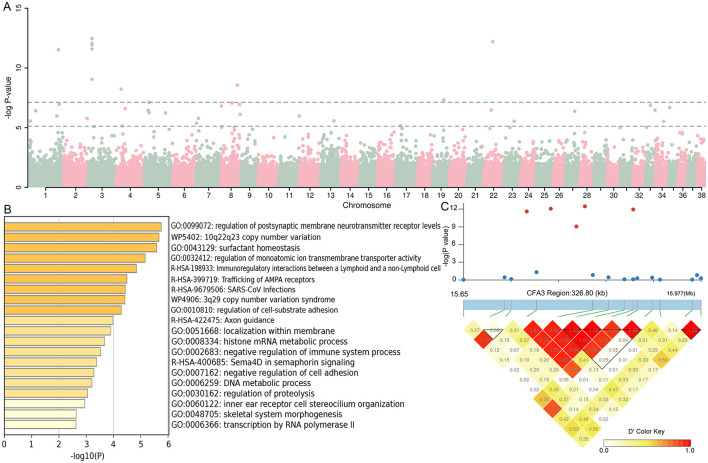
Genomic association analysis between 12 infected dogs and 788 dogs with brachycephalic and mesocephalic characteristics. **(A)** Manhattan map of GWAS. **(B)** The function of the 180 genes enrichment analysis. **(C)** LD block analysis of the two regions on CFA3.

GWAS analysis of 12 cherry-eyed affected dogs vs. 788 brachycephalic and mesocephalic dogs showed 40 SNPs exceeding the threshold of significance at the chromosomal level and 11 SNPs exceeding the significance threshold at the genomic level (Figure 3A). SNPs exceeding the significance threshold at the chromosomal level and their upstream and downstream 5Kb regions were genetically annotated, and a total of 180 genes (Supplementary Table S2) were identified and subjected to functional enrichment analysis, which showed that these genes were involved in multiple biological functions (Figure 3B). Similar to the above results, we found five SNPs in the CFA22:15627075-15983629 bp region that exceeded the significance threshold at the genome level, and chaining analysis was performed on this region, and these five SNPs were found to be strongly chained (Figure 3C).

**Figure 4:**
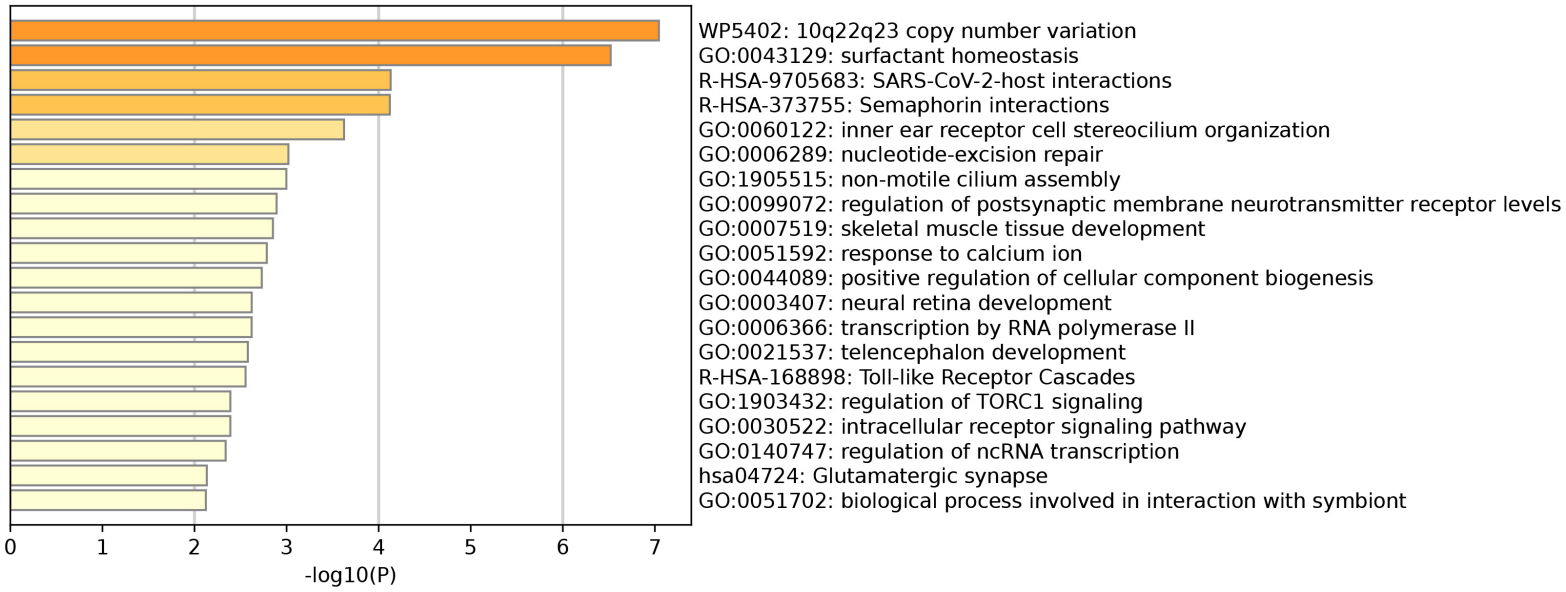
GO term “GO: 0003407” pathway involved in neuroretinal development.

The results showed that a total of 104 annotated genes were identified in both GWAS analyses, and we further performed functional enrichment analysis on these 104 genes, which showed that there were multiple genes involved in neurodevelopment and other processes. It is worth mentioning that the GO term “GO:0003407” pathway is involved in the neural retinal development pathway (Figure 4), and the genes involved in the high pathway include *DIO3* and *TTC8* (23–25). Therefore, these 104 genes were analyzed in depth, and 33 were found to be associated with eye development or eye diseases (Supplementary Table S3).

## 4 Discussion

As human companion animals, dogs have the advantages of a large number of naturally occurring genetic diseases with similar body sizes and diets, the same living environment, and a unique population structure that provides high-quality genome-wide reference sequences. Moreover, dog and human genomes have a high degree of similarity (75%), making them ideal animal models for studying spontaneous genetic diseases in humans (26–29). According to the online Human Mendelian Inheritance Database (https://omim.org/home/), as of 2020, ~700 dogs with monogenic diseases or complex traits have been identified, of which at least 230 have known disease-causing mutations and 340 dogs are potential models for human disease (30). The number of spontaneous genetic diseases in other domesticated animals was significantly lower than that in dogs. A large number of dogs with spontaneous genetic diseases can be used as animal models for research, providing more possibilities for studying and treating human genetic diseases (31–33). Third eyelid gland prolapse is a relatively common disease in domesticated animals and has diverse causes (34). A total of 1,802 cases of third eyelid prolapse were identified in 905,543 dogs, with an annual prevalence of 0.20% (35). However, most studies on third eyelid gland prolapse in domestic dogs have focused on the treatment of this disease and the underlying genetic mechanisms have not yet been analyzed. The present study aimed to contribute to the understanding of ocular tumors in humans by analyzing the mechanism of third eyelid gland prolapse.

In this study, we performed GWAS analysis based on 12 dogs with cherry eyes and brachycephalic dogs and identified SNPs. Their upstream and downstream regional gene annotations revealed that some genes are indeed involved in ocular development or are associated with ocular diseases in brachycephalic dogs, *NR2F1* may be associated with optic nerve atrophy syndrome. GWAS was also performed on both brachycephalic and mesocephalic dogs, and 33 genes were found to be associated with ocular development or disease in breeds with different cranial structures, including genes associated with diabetic retinopathy, such as *SYT3, MYBPC2*, and *POLD1* (36, 37). These findings are important for exploring the causative genes of third eyelid protrusions in dogs.

In addition to common dog diseases, dogs can also suffer from diseases similar to humans, such as tumors like eye tumors and psychiatric disorders like autism, and studies have shown a strong association between *SHANK3* and autism in humans (38, 39). By analyzing the whole genome sequence of domestic dogs, it was found that the structure of the *SHANK3* gene in dogs was similar to that of humans. Therefore, the researchers carried out gene editing in dogs, and the results showed that the mutant dogs all showed different degrees of abnormal motor ability, repetitive stereotypy, as well as the presence of severe social abnormalities and cognitive deficits between dogs and dogs, and between dogs and humans, and other clinical symptoms of autism. There are no ideal treatments for fatal human genetic diseases, mainly because of the limited research on the natural history of the disease, insufficient understanding of the correlation between genotype and disease, lack of effective alternative markers, and lag in translational research on effective animal models (40). Therefore, it is necessary to study the natural occurrence and molecular mechanisms of human genetic diseases and evaluate the safety and effectiveness of gene therapy in large animal models (41).

In this study, although we did not fully elucidate the pathogenesis of third eyelid gland prolapse in dogs, this limitation may be attributed to the small sample size or the genetic diversity of the breeds collected. However, we genetically annotated SNPs and their upstream and downstream 5Kb regions associated with canine third eyelid gland ptosis that exceeded the significance threshold at the chromosome level and identified some genes associated with ocular development or disease. This represents a significant step forward in understanding the pathogenesis of third eyelid gland prolapse in dogs.

## Data Availability

The datasets presented in this study can be found in online repositories. The names of the repository/repositories and accession number(s) can be found in the article/Supplementary material.

## Supplementary material

Supplementary Table 1 240 genes identified by GWAS between 12 dogs with cherry eye and 653 brachycephalic dogs.

Supplementary Table 2 180 genes identified by GWAS from 12 cherry eyed dogs and 788 brachycephalic and mesocephalic dogs.

Supplementary Table 3 Total of 33 genes were identified that were associated with eye development or disease.
